# Multivariate Autoregressive Modeling and Granger Causality Analysis of Multiple Spike Trains

**DOI:** 10.1155/2010/752428

**Published:** 2010-04-29

**Authors:** Michael Krumin, Shy Shoham

**Affiliations:** Faculty of Biomedical Engineering, Technion—Israel Institute of Technology, 32000 Haifa, Israel

## Abstract

Recent years have seen the emergence of microelectrode arrays and optical methods allowing simultaneous recording of spiking activity from populations of neurons in various parts of the nervous system. The analysis of multiple neural spike train data could benefit significantly from existing methods for multivariate time-series analysis which have proven to be very powerful in the modeling and analysis of continuous neural signals like EEG signals. However, those methods have not generally been well adapted to point processes. Here, we use our recent results on correlation distortions in multivariate Linear-Nonlinear-Poisson spiking neuron models to derive generalized Yule-Walker-type equations for fitting ‘‘hidden” Multivariate Autoregressive models. We use this new framework to perform Granger causality analysis in order to extract the directed information flow pattern in networks of simulated spiking neurons. We discuss the relative merits and limitations of the new method.

## 1. Introduction

The analysis of multivariate neurophysiological signals at the cellular (spike trains) and population scales (EEG/MEG, LFP, and ECOG) has developed almost independently, largely due to the mathematical differences between continuous and point-process signals. The analysis of multiple neural spike train data [1] has gained tremendous relevance recently with the advent and widespread application of arrays of microelectrodes in both basic and applied Neurosciences. Furthermore, emerging optical methods for network activity imaging [2] and control [3] are likely to further compound this growth.

 Currently, the analysis of multichannel spike trains is still largely limited to single-channel analyses, to bivariate cross-correlation and metric-space analyses [4], and to spike train filtering (“decoding”). In contrast, much of EEG/MEG time series analysis has revolved around linear and nonlinear models and analyses that are essentially multivariate, most prominently the multivariate autoregressive (MVAR) model. The MVAR framework is associated with a powerful set of time- and frequency-domain statistical tools for inferring directional and causal information flow based on Granger's framework [5], including linear and nonlinear Granger causality, directed transfer function, directed coherence, and partial directed coherence (see [6–8] for reviews). Scattered attempts at applying this general framework to neural spike trains have relied on smoothing the spike trains to obtain a continuous process that can be fit with an MVAR model [9–12]. This approach has the clear disadvantage of being highly kernel dependent and of introducing unwanted distortions. The inability to estimate multivariate autoregressive models for spike trains has recently motivated Nedungadi et al. [13] to develop an alternative nonparametric procedure for computing Granger causality based on spectral matrix factorization (without fitting the data with an autoregressive model). 

 The purpose of this paper is to bridge this divide in neurophysiological data analysis by introducing a correlation-distortion-based framework for applying multivariate autoregressive models to multichannel spike trains. The primary aim of making this connection is to enable direct identification of causal information flow among populations of neurons using the powerful Granger causality analyses, which have been tried and tested in numerous studies of continuous neural signals. The framework is based on our recent analytical results [14, 15] on correlation distortions in (multiple) Linear-Nonlinear-Poisson (LNP) models when the inputs are white Gaussian noise processes and the nonlinearities are exponential, square, or absolute values. The essential idea in this approach is that the nonlinearity (which produces the firing rates) systematically distorts the correlation structure of the correlated Gaussian outputs of the linear stage, and that the spike trains carry essentially the same expected correlation structure. By deriving formulas for these distortions, we were able to generate synthetic spike trains with a fully-controllable correlation structure by choosing FIR linear kernels that “predistort” the Gaussian processes to cancel out the subsequent distortion. Such spike trains can be applied, for example, in pattern photo-stimulation of synthetic input activity onto a neuron, or for controlling neuron populations in artificial neuroprosthetic interfaces [3, 16]. Although we noted in [14] that the linear stage could generally have a recursive MVAR structure, the required estimation steps were not presented or tested. 

 The remainder of the paper is organized as follows. Section 2 introduces the methods used for generating the spike trains used in this paper and for evaluating statistical significance. In Section 3 we present and evaluate the procedure for estimating the MVAR-nonlinear-Poisson model. In Section 4 we provide an overview of linear Granger causality analyses and apply them to estimating information flow in bi- and trivariate spike trains. In Section 5 we conclude by discussing the new framework's relation to previous work, and its prospects and limitations.

## 2. Methods

### 2.1. Synthetic Spike Train Generation

Spike trains were generated in two different ways in order to mimic two basic scenarios encountered in neural data recordings: distributed population activity with relatively wide correlation functions and local network with directly interconnected neurons. 

 Population activity was simulated using a Linear-Nonlinear-Poisson (LNP) generative neural model with a multichannel linear stage modeled by a Multivariate Autoregressive model (see Section 3). Causal connectivity structures were generated by choosing appropriate coefficients for the MVAR model (details provided for each example in Section 4). The desired mean firing rates and firing rate variability were obtained by adjusting the parameters of the nonlinearity [14].

**Figure 1:**
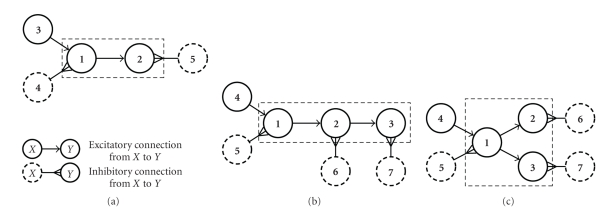
Structure of simulated networks (a)–(c) of IF neurons. Morphologies of the three networks simulated to provide data for the analysis of realistic local networks (see Figure 4 for the results). Granger causality analysis was performed on the subnetworks marked by dashed boxes. The additional neurons were used to balance excitatory and inhibitory input to the analyzed cells.

 Local networks of directly interconnected neurons were simulated using integrate-and-fire (IF) neuron models proposed by Izhikevich [17] with various interconnection schemes. Routines based on freely available code from the ModelDB [18, 19] database (accession number 115968) were used to generate this network activity. This approach provided networks of realistic spiking neurons with no assumptions of Poisson firing or LNP-based activity. For the three examples used in this work, three different networks were simulated with the connectivity structures depicted in Figure 1. 

### 2.2. Surrogate Data Generation

To perform a statistical test on the results of the proposed method as part of Granger causality analyses, surrogate datasets were generated. The surrogate data was generated by nullifying one causal connection (coefficient) at a time in the estimated underlying MVAR model of the linear stage. This new MVAR model (with one artificially “broken” connection) was used for generating spike trains with no direct causal relation between the two tested channels. Each of these spike trains was then analyzed by the proposed method for Granger causality. The resultant coefficients (6) or (8) provided a “null” distribution, to which the corresponding coefficients calculated from the real data were compared.

## 3. Identifying an MVAR-Nonlinear-Poisson Model

We consider the problem of identifying a *p*-dimensional multivariate (vector) autoregressive model of order *m*:

(1) $$x\left(n\right)=-\overset{m}{\underset{k=1}{\sum }}A\left(k\right)\cdot x\left(n-k\right)+w\left(n\right)$$

from observations of Poisson spike trains whose rate depends nonlinearly on **x**(*n*):

(2) $$\lambda_{i}\left(n\right)=f\left(\mu_{i}+\sigma_{i}\cdot x_{i}\left(n\right)\right).$$

The matrices *A*(*k*) are *p* × *p* coefficient matrices, each corresponding to a specific lag, and **w** is a zero-mean Gaussian noise process with covariance matrix ∑.

 The parameters of an MVAR model (*A*(*k*)) can be estimated directly from the autocorrelation function of its output *R*_**x**_(*k*) = *E*[**x**(*n*) · **x** ^*T*^(*n* + *k*)] by solving the multivariate Yule-Walker equations [8]:

(3) $$\overset{m}{\underset{k=1}{\sum }}A\left(k\right)\cdot R_{x}\left(i-k\right)=-R_{x}\left(i\right),\;1\leq i\leq m.$$

How can this framework be adapted to our case? Although the correlation matrices *R*_*x*_(*k*) are not directly measurable, they can be indirectly estimated from the correlation matrices and expectations of the firing rates *λ* for certain choices of the nonlinearity *f*. These “predistortion” relationships were derived in [14] for exponential, square and absolute-value transformations by considering the effect of these nonlinearities on pairs of correlated, normally distributed random variables. For the case of doubly-stochastic Poisson processes, the spike-train correlation structure *R*_Δ*N*_(*τ*) is identical to that of the firing rates, except at zero lag [14, 20]: *R*_Δ*N*_(*τ*) = *R*_*λ*_(*τ*) + *δ*(*τ*)*E*(*λ*).

The parameter estimation algorithm is summarized in Algorithm 1 for the exponential and square nonlinearities (the detailed derivation and formulas of absolute-value pre-distortions appear in [14]). 

The main algorithm assumes that the model order *m* is known. Several different criteria for automatic determination of an “optimal” model order *m* have been developed. In our implementation we determined the model order by minimizing the Bayesian Information Criterion (BIC):

(4) $$\text{BIC}\left(m\right)=2\log\;\left[\det\;\left(\tilde{\Sigma }\right)\right]+\frac{2p^{2}m\cdot \log\;N_{\text{total}}}{N_{\text{total}}},$$

where *N*_total_ is the total number of data points and the prediction error covariance matrix $\tilde{\Sigma }$ is given by

(5) $$\tilde{\Sigma }=R\left(0\right)+\overset{m}{\underset{k=1}{\sum }}A\left(k\right)\cdot R_{x}\left(k\right).$$

**Figure 2:**
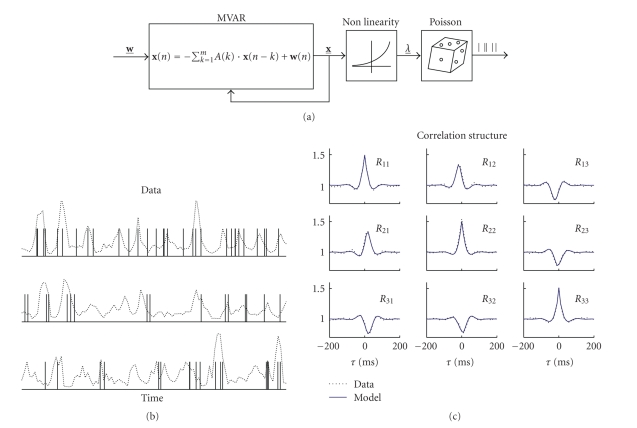
Fitting multiple spike trains with an MVAR-Nonlinear-Poisson model. (a) Schematic representation of the model. (b) Rate processes *λ*(*t*) and corresponding Poisson spike trains. (c) Correlation structures of the original spike trains and the estimated model.

Figure 2 presents an illustrative example of an MVAR-Nonlinear-Poisson model (Figure 2(a)) of order 3 estimated from three correlated spike trains. The correlations between the three processes, which can be qualitatively appreciated from the firing rates (Figure 2(b)), are accurately captured by the estimated model (Figure 2(c)).

## 4. Granger Causality Analysis

Granger causality is based on the general concept due to Norbert Wiener that a causal influence should manifest in improving the predictability of the driven process when the driving process is observed. A measurable reduction in the unexplained variance of the driven process (say *x*_2_(*n*)) as a result of inclusion of the causal (driving) process (say *x*_1_(*n*)) in linear autoregressive modeling marks the existence of a causal influence from *x*_1_(*n*) to *x*_2_(*n*) in the time domain [5]. Pairwise linear Granger causality in the time domain is defined as

(6) $$F_{x_{1}\to x_{2}}=\ln\;\frac{\Sigma_{1}}{\Sigma_{2}},$$

where Σ_1_ is the unexplained variance (prediction error covariance) of *x*_2_(*n*) in its own autoregressive model, whereas Σ_2_ is its unexplained variance when a joint MVAR model for both *x*_2_(*n*) and *x*_1_(*n*) is constructed. It is expected that *F*_*x*_1_→*x*_2__ > 0 when *x*_1_(*n*) influences *x*_2_(*n*), and *F*_*x*_1_→*x*_2__ = 0 when it does not. In practice, *F*_*x*_1_→*x*_2__ is compared to a threshold value, which can be determined using a variety of methods (typically using surrogate data or by shuffling channels).

**Figure 3:**
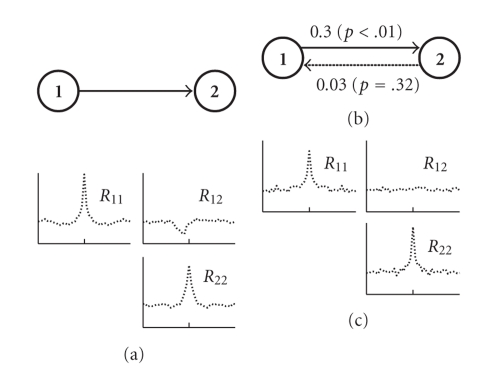
Granger causality analysis in a 2D case. (a) Schematic of the original model and its observed correlation structure. (b) Granger causality analysis of the spike trains revealed a significant causal connection from cell 1 to cell 2. The numbers represent the pairwise linear Granger causality coefficients for each connection and their *P*-values (in parenthesis). (c) Correlation structure of the model used as a “null” for the statistical test, with no connection between cell 1 and cell 2.

 To evaluate the Granger analysis framework, we first tested its ability to estimate the causality structure in a system of two coupled synthetic spike trains with unidirectional influence (Figure 3). The structure of the MVAR model used to generate the data (Figure 3(a)) was

(7) $$\underline{x}\left(n\right)=\left[\begin{array}{cc} \frac{1}{2} & 0\\ 0 & \frac{1}{2} \end{array}\right]\underline{x}\left(n-1\right)+\left[\begin{array}{cc} 0 & 0\\ -\frac{1}{2} & 0 \end{array}\right]\underline{x}\left(n-2\right)+\underline{w}\left(n\right).$$

We simulated a 10-minute-long dataset where the mean firing rate of the spike trains was 20 Hz (set by adjusting the exponent's parameters). The strength of each connection was calculated using (6), and their statistical significance was tested by applying the same calculation scheme to surrogate data where the tested connection was removed (for details on generating surrogate data see Section 2).

Pairwise causal analyses are important, but cannot distinguish, for example, between direct and indirect influences in more elaborate connectivity schemes, such as trivariate networks [8]. To address inference in this more complex scenario, we can perform a series of “leave-one-out” analyses, using the multivariate extension of the linear Granger causality index [7, 8]. For example, to assess the *direct* influence exerted by *x*_*m*_ on *x*_*n*_, we use

(8) $$F_{x_{m}\to x_{n}}=\ln\;\frac{\Sigma_{x_{n}\;|\;x_{1}\cdots x_{m-1},x_{m+1}\cdots x_{p}}}{\Sigma_{x_{n}\;|\;x_{1}\cdots x_{p}}}.$$

**Figure 4:**
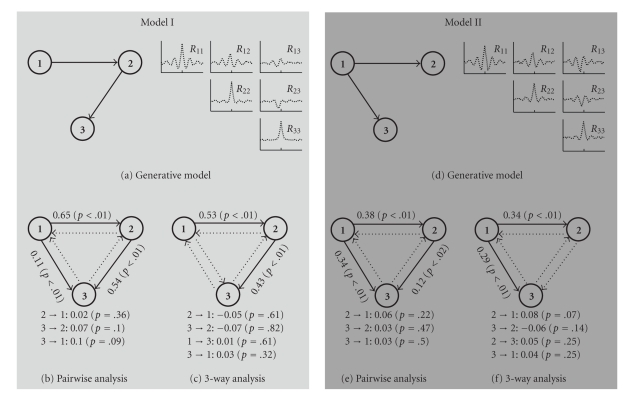
Granger causality analysis of different 3D models. (a, d) Schematic of the models and their correlation structures. (b, e) Pairwise Granger causality analysis (using (6)) is incapable of distinguishing between direct and indirect connections, and three causal connections are deemed significant. (c, f) Three dimensional Granger causality analysis reveals the connectivity structure that fits the original models. Granger causality coefficients were calculated using (8). The statistical test was performed using surrogate data.

To test this approach (Figure 4) we applied it to synthetic data originating from 10-minute-long datasets (20 Hz average rate) synthetically generated from two different MVAR models that represent two basic triple-unit activity examples. The first one (Figures 4(a)–4(c)) models sequential connection and was modeled by

(9) $$\begin{array}{cc} \underline{x}\left(n\right) & =\left[\begin{array}{ccc} \frac{1}{2} & 0 & 0\\ \frac{1}{2} & \frac{1}{2} & 0\\ 0 & 0 & \frac{1}{2} \end{array}\right]\underline{x}\left(n-1\right)+\left[\begin{array}{ccc} 0 & 0 & 0\\ 0 & 0 & 0\\ 0 & -\frac{1}{2} & 0 \end{array}\right]\underline{x}\left(n-2\right)\\ & \;+\left[\begin{array}{ccc} -\frac{1}{2} & 0 & 0\\ 0 & 0 & 0\\ 0 & 0 & 0 \end{array}\right]\underline{x}\left(n-3\right)+\underline{w}\left(n\right). \end{array}$$

The second case (Figures 4(d)–4(f)) is a “common input” example, where unit #1 drives the activity of units #2 and #3 with different time delays. This example was modeled by

(10) $$\begin{array}{cc} \underline{x}\left(n\right) & =\left[\begin{array}{ccc} \frac{1}{2} & 0 & 0\\ \frac{1}{2} & \frac{1}{2} & 0\\ 0 & 0 & \frac{1}{2} \end{array}\right]\underline{x}\left(n-1\right)+\left[\begin{array}{ccc} 0 & 0 & 0\\ 0 & 0 & 0\\ 0 & 0 & 0 \end{array}\right]\underline{x}\left(n-2\right)\\ & \;+\left[\begin{array}{ccc} -\frac{1}{2} & 0 & 0\\ 0 & 0 & 0\\ -\frac{1}{2} & 0 & 0 \end{array}\right]\underline{x}\left(n-3\right)+\underline{w}\left(n\right). \end{array}$$

**Figure 5:**
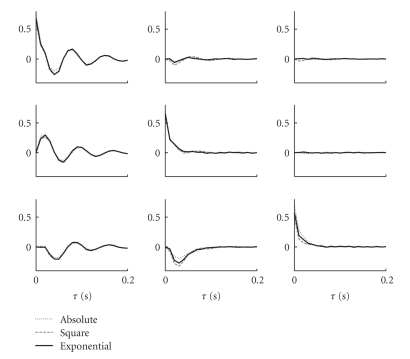
Nonlinearity mismatch has a minor effect on the estimation of the MVAR model. MVAR-N model was estimated from spike trains generated using different nonlinearities (absolute value (dotted line), square (dashed line), or exponential (solid line)). Estimation was done using the exponential nonlinearity in all the cases. The impulse response of the linear MVAR model was affected only slightly by such nonlinearity mismatch. Kernel in row *#m *and in column* #n* represents the response of channel *#m* on impulse input to channel *#n*.

To test the robustness of the MVAR-N-P model estimation under a nonlinearity mismatch, we simulated Model I from Figure 4 using a square and an absolute value nonlinearity and estimated the model assuming an exponential nonlinearity as before. As can be seen in Figure 5, the estimated MVAR models are extremely similar to the model estimated without the mismatch: *ρ* = 0.995 ± 0.004, *ρ* = 0.993 ± 0.006, respectively, for the nonzero kernels (each model is illustrated by its respective impulse responses). 

**Figure 6:**
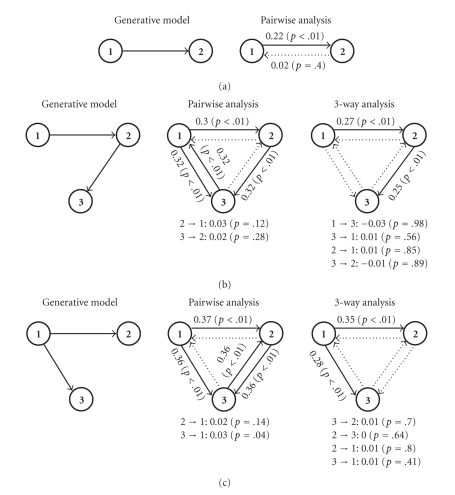
Granger causality analysis of realistic Izhikevich-type networks of neurons. Granger causality analysis was applied to reconstruct the connectivity structure in three basic architectures—see Figure 1 for the full structure of the simulated populations. (a) Two cells with unidirectional causal connection. (b) Three cells with sequential connection. (c) Three cells with “common input” interaction. Note that in examples (b) and (c) the reduced 2-dimensional models do not reconstruct correctly the 3D causal relations, as expected.

Finally, we tested the method on data that comes from simulations of *realistic* local network activity. The spike trains were simulated using interconnected networks of integrate-and-fire neurons [17]. Examples similar to those presented in Figures 3 and 4 were analyzed by the proposed approach based on the MVAR-Nonlinear-Poisson model. Exponential nonlinearity was used as it is more flexible in capturing correlation structures than the other two nonlinearities [14]. The method successfully determines the connectivity pattern in all three examples, even though the spike trains are far from being Poissonian and therefore cannot be generated by an LNP model (Figure 6). 

We note that, as a result of absolute and relative refractory periods of non-Poisson spiking, the correlation structure has strong negative peaks that cannot be directly captured by the MVAR models used in our framework. To fit a stable and representative MVAR model to these spike trains, we applied a basic regularization procedure to their correlation structure that consisted of truncating the negative peaks in the auto- and cross-covariance functions and adding a diagonal matrix to the correlations matrix (in order to get a positive semidefinite correlation matrix).

## 5. Discussion

In this paper, we introduced a new method for modeling multineuron spike train data, and its application to the identification of information flow structure among interacting neuronal populations. The identification of neural systems from multineuron spike train data can be used for experimental inference of underlying network connections [22–27], or more generally of “effective” connectivity. It is also indirectly related to nonparametric methods for identifying high-order synchronous interactions [28–31], and metrics of (dis)similarity between spike trains [4, 32–36]. In our approach, the data is fit to a model based on an underlying MVAR process with Gaussian statistics which is nonlinearly transformed to firing rates that modulate Poisson spike trains. Our approach thus departs from the classical model-based identification of multivariate spike train data which assumes a specific, biophysically-motivated, linear or nonlinear interaction scheme between neurons [22–27]. In our approach, there is no explicit modeling of the interaction exerted through the spike trains, but rather the modulating processes interact through the multivariate recursive structure of the MVAR. In practice, the strict assumptions of neural-interaction models are challenged by the dramatic under-sampling of a large population in real neural recordings, as well as by oscillations and modulations exerted by unmodeled elements. Recently, this “classical” approach has been generalized into generalized linear models (GLMs) that include modulation by a dynamic stimulus or behavior [37–39], which in our framework can be done by adding an additional external input or set of inputs (and thus moving from an AR to an ARX model). In addition, the new framework can easily be extended to analyze mixed datasets containing both spiking and continuous neurophysiological (and behavioral) data, as well as to time-varying (nonstationary) scenarios using the MVAR framework and its standard adaptive extensions.

Rather than explicitly modeling neuron-to-neuron interactions, our approach benefits from the MVAR-based Granger causality framework for inferring directed information flow using the concept of increased predictability of one time series when another is observed. While we have only illustrated the use of linear Granger causality, this framework now includes a number of different statistical measures that can be used for inference, including directed transfer function, directed coherence, and partial directed coherence. A thorough overview of these methods [6–8] is clearly outside the scope of the current paper; however, we note that their ability to infer cortical network connectivity patterns has been systematically tested and compared in a number of studies (e.g., see [40]). It is important to note that a major difference between our approach and “classical” MVAR models is that the MVAR model here is hidden and only indirectly observed through the noisy spike trains. As a result, it was crucial that both the MVAR parameter estimates and the prediction errors can both be estimated from auto- and cross-correlations equations (3) and (5).

 Finally, we note that the proposed framework has some limitations that could potentially benefit from additional work. The LNP nonlinearity is required in order to transform the Gaussian processes into nonnegative stochastic intensities (rates) that modulate the Poisson processes. Having to select a specific nonlinearity is clearly a shortcoming of this approach versus the complete generality (and uniqueness) of the MVAR framework in the context of continuous processes. Secondly, we note that when the MVAR-N-P is used as a generative model for spike trains, the doubly-stochastic Poisson processes that are produced have a larger-than-Poisson dispersion (variance), which may not always be desirable. This limitation can be addressed by considering alternative models for the transformation from a Gaussian process to spike trains. For example, the single-stochastic processes analyzed by Macke et al. [41] produce spikes deterministically whenever a Gaussian process is higher than a threshold value, while Tchumatchenko et al. [42] analyzed spike trains produced during threshold crossings of Gaussian processes (both solutions can only be numerically inverted).

## Figures and Tables

**Algorithm 1:**
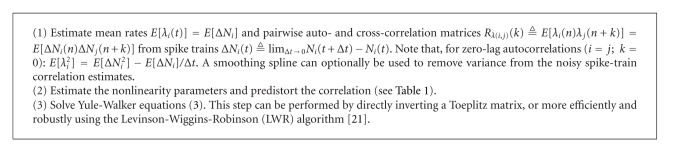
Algorithm outline.

**Table 1:** 

*λ*_*i*_(*t*)	Exponential	Square
	exp (*μ*_*i*_ + *σ*_*i*_ *x*_*i*_(*t*))	(*μ*_*i*_+*σ*_*i*_ *x*_*i*_(*t*))^2^

*μ*_*i*_	$\ln\;\left(\frac{E^{2}\left[\lambda_{i}\right]}{\sqrt{E\left[\lambda_{i}^{2}\right]}}\right)$	$\sqrt[{4}]{\frac{3}{2}E^{2}\left[\lambda_{i}\right]-\frac{E\left[\lambda_{i}^{2}\right]}{2}}$

*σ*_*i*_	$\sqrt{\ln\;\left(\frac{E\left[\lambda_{i}^{2}\right]}{E^{2}\left[\lambda_{i}\right]}\right)}$	$\sqrt{E\left[\lambda_{i}\right]-\sqrt{\frac{3}{2}E^{2}\left[\lambda_{i}\right]-\frac{E\left[\lambda_{i}^{2}\right]}{2}}}$

*R*_**x**(*i*,*j*)_(*k*)	$\frac{\ln\;\left(R_{\lambda (i,j)}\left(k\right)/E\left[\lambda_{i}\right]E\left[\lambda_{j}\right]\right)}{\sigma_{i}\sigma_{j}}$	$\frac{-\mu_{i}\mu_{j}+\sqrt{\left(R_{\lambda (i,j)}\left(k\right)-E\left[\lambda_{i}\right]E\left[\lambda_{j}\right]\right)/2+\mu_{i}^{2}\mu_{j}^{2}}}{\sigma_{i}\sigma_{j}}$
